# Pretreatment neutrophil-to-lymphocyte ratio is correlated with response to neoadjuvant chemotherapy as an independent prognostic indicator in breast cancer patients: a retrospective study

**DOI:** 10.1186/s12885-016-2352-8

**Published:** 2016-05-19

**Authors:** Yi Chen, Kai Chen, Xiaoyun Xiao, Yan Nie, Shaohua Qu, Chang Gong, Fengxi Su, Erwei Song

**Affiliations:** Department of Breast Tumor Center, Sun Yat-sen Memorial Hospital, Sun Yat-sen University, 107# Yanjiang West Road, Guangzhou, China; Department of Ultrasound, Sun Yat-sen Memorial Hospital, Sun Yat-sen University, 107# Yanjiang West Road, Guangzhou, China; Guangdong Provincial Key Laboratory of Malignant Tumor Epigenetics and Gene Regulation, Sun Yat-sen Memorial Hospital, Sun Yat-sen University, 107# Yanjiang West Road, Guangzhou, China; Collaborative Innovation Center for Cancer Medicine, Guangzhou, China

**Keywords:** Breast cancer, Neutrophil to lymphocyte ratio, Pathologic complete response, RFS, BCSS, Neoadjuvant chemotherapy

## Abstract

**Background:**

A high neutrophil-to-lymphocyte ratio (NLR) may be related to increased mortality in patients with lung, colorectal, stomach, liver, and pancreatic cancer. To date, the utility of NLR to predict the response to neoadjuvant chemotherapy (NAC) has not been studied. The aim of our study was to determine whether the NLR is a predictor of response to NAC and to investigate the prognostic impact of the NLR on relapse-free survival (RFS) and breast cancer-specific survival (BCSS) in patients with breast cancer who received NAC.

**Methods:**

We retrospectively studied patients who received NAC and subsequent surgical therapy for stage II–III invasive breast carcinoma at Sun Yat-sen Memorial Hospital between 2001 and 2010. The correlation of NLR with the pathological complete response (pCR) rate of invasive breast cancer to NAC was analyzed. Survival analysis was used to evaluate the predictive value of NLR.

**Results:**

A total of 215 patients were eligible for analysis. The pCR rate in patients with lower pretreatment NLR (NLR < 2.06) was higher than those with higher NLR (NLR ≥ 2.06) (24.5 % vs.14.3 %, *p* < 0.05). Those patients with higher pretreatment NLR (NLR ≥ 2.1) had more advanced stages of cancer and higher disease-specific mortality. Through a multivariate analysis including all known predictive clinicopathologic factors, NLR ≥ 2.1 was a significant independent parameter affecting RFS (HR: 1.57, 95 % CI: 1.05-3.57, *p* < 0.05) and BCSS (HR: 2.21, 95 % CI: 1.01-4.39, *p* < 0.05). Patients with higher NLR (NLR ≥ 2.1) before treatment showed significantly lower relapse-free survival rate and breast cancer-specific survival rate than those with lower NLR (NLR <2.1) (log-rank *p* = 0.0242 and 0.186, respectively).

**Conclusions:**

Pretreatment NLR < 2.06 is associated with pCR rate, suggesting that NLR may be an important factor predicting the response to NAC in breast cancer patients. NLR is an independent predictor of RFS and BCSS in breast cancer patients with NLR ≥ 2.1 who receive NAC. We suggest prospective studies to evaluate NLR as a simple prognostic test for breast cancer.

**Electronic supplementary material:**

The online version of this article (doi:10.1186/s12885-016-2352-8) contains supplementary material, which is available to authorized users.

## Background

Neoadjuvant therapy was initially used in patients with inoperable locally advanced tumors. Neoadjuvant and adjuvant administration of chemotherapy are equivalent in terms of overall survival [1–4]. Neoadjuvant chemotherapy used in patients with initially operable tumors is superior for increasing the chance of achieving breast-conserving surgery, evaluating the susceptibility of chemotherapy drugs and assessing the response to chemotherapy. Patients with a pCR after neoadjuvant chemotherapy have better disease-free survival. The FDA recently granted accelerated approval for pertuzumab in combination with trastuzumab and docetaxel as neoadjuvant treatment for patients with Her-2-positive breast cancer as a result of the significant improvement in pCR in patients. pCR has become an important parameter in the approval of a new drug by FDA, so it is important to find a clinical pathological indicator to predict pCR in advance.

Predictive factors of the response to neoadjuvant chemotherapy include tumor size, pathology subtype, and differentiation as well as expression of estrogen receptor (ER), progesterone receptor (PR), human epidermal growth factor receptor 2 (HER2) and KI67 [5]. There is increasing evidence that the neutrophil to lymphocyte ratio is associated with long-term outcomes, so this ratio has gained much interest, with several studies over the last 5 years investigating its role in predicting long-term outcomes in various cancer populations, including lung, colorectal, stomach, liver, and pancreatic cancer [6–10]. Based on studies that show the association between high NLR and increased mortality in breast cancer [11–13], we suggest that NLR could be an important predictor of the response to neoadjuvant chemotherapy as an inflammatory indicator. The aim of the present study was to investigate the association of NLR with pCR in patients who received neoadjuvant chemotherapy and the prognostic value of NLR in view of RFS and BCSS.

## Methods

### Data collections

We retrospectively identified 347 patients who were diagnosed with primary breast cancer and received NAC at Sun Yat-sen Memorial Hospital between January 2001 and June 2010. The study was given ethical approval with Ethical Committee of Sun Yat-sen Memorial Hospital and all the patients had given written informed consent. The inclusion criteria were as follows: (1) female aged 18 to 70, whose expected survival time was more than 12 months; (2) clinical stage II or III; (3) diagnosed with primary breast cancer by core needle biopsy before NAC; (4) received 3 cycles or more than 3 cycles of NAC after diagnosis and underwent curative-intent surgery such as breast-conserving surgery or modified radical mastectomy. Patients with ductal carcinoma in situ with or without microinvasion, patients with missing information on pathologic or laboratory results, and patients who were lost to follow-up were excluded. We also excluded patients with stage IV breast cancer or inflammatory breast cancer; patients who were diagnosed preoperatively with systemic inflammatory or chronic disease, such as systemic lupus erythematosus (SLE), liver cirrhosis, or end-stage renal disease; and patients with pregnancy-related breast cancer.

Of these, 215 patients met the inclusion criteria. Medical records were reviewed to find data on each patient’s medical history, age, sex, chemotherapy regimen of NAC, chemotherapy cycles of NAC, surgical method, pathologic results (such as histologic type, tumor size, histological grade, and lymph node status (number of positive lymph nodes and all lymph nodes if axillary lymph nodes were dissected), hormonal status, and HER2 receptor status), and laboratory data (including C-reactive protein (CRP)). The tumor size (T stage), lymph node status (N stage), presence of metastasis (M stage) and the American Joint Committee on Cancer (AJCC) stage for each patient were obtained by reviewing the cancer registry data. T stage, N stage and M stage before and after surgery are according to AJCC [14].

We used taxane-based and/or anthracycline-based chemotherapy regimens in neoadjuvant settings every 21 days: epirubicin and cyclophosphamide (EC, E: 90 mg/m^2^, C: 600 mg/m^2^); docetaxel and cyclophosphamide (TC, T: 100 mg/m^2^, C: 600 mg/m^2^); docetaxel, epirubicin and cyclophosphamide (TEC, T: 75 mg/m^2^, E: 90 mg/m^2^, C: 500 mg/m^2^); and docetaxel, carboplatin and trastuzumab (TCH, T: 75 mg/m^2^, C: AUC = 5, H: 8 mg/kg followed by 6 mg/kg). Trastuzumab was added if the tumor was positive for HER2 (but only 39 % of patients with Her-2 positive had taken Herceptin as adjuvant treatment because of the high price). Neoadjuvant therapy, surgery, radiotherapy and endocrine therapy were provided to patients according to National Comprehensive Cancer Network (NCNN) guidelines [14].

In all the patients, a routine blood test of peripheral vein blood was performed immediately after breast cancer diagnosis and before the initiation of any treatment modality (pretreatment NLR). NLR was calculated as the ratio of absolute neutrophil count to absolute lymphocyte count in this blood sample. A routine blood test was also taken right before surgery (approximately 2-weeks after the last cycle of NAC) so that the change in NLR from before to after NAC could be calculated.

### Pathology

We graded tumors according to the Scarff-Bloom-Richardson [15] scheme. ER and PR status were assessed by immunohistochemistry. ER and PR assays were considered positive if there were at least 1 % positive tumor nuclei in the sample on testing in the presence of expected reactivity of internal (normal epithelial elements) and external controls [16]. HER2 status was assessed by immunohistochemistry and/or fluorescent in situ hybridization (FISH). It was considered positive if the score was 3 with immunohistochemistry or there were at least 2.2 times as many HER2 signals as CEP 17 signals in the tumor cells.

Molecular subtype was divided into 4 groups according to the immunohistochemical staining for ER, PR, HER2 and KI67 [17]: luminal A subtype, ER-positive and/or PR-positive and HER2-negative, KI67 < 14 %; luminal B subtype, ER-positive and/or PR-positive and HER2-positive, or ER-positive and/or PR-positive and HER2-negative, KI67 ≥ 14 %; HER2-enriched subtype, ER- and PR-negative with positive HER2; triple-negative tumors, ER-negative, PR-negative and HER2-negative.

### Assessing chemotherapy response

Clinical remission was assessed for primary tumors through physical examination and ultrasonic measurement after all cycles of NAC before surgery. The response to neoadjuvant chemotherapy was according to the NSABP criteria [18] for therapeutic effect evaluation: clinical complete response (cCR): the absence of clinical evidence of tumor in the breast; clinical partial response (cPR): the product of the two largest perpendicular diameters of the breast tumor had decreased by 50 % or more; stable disease (cS): patients whose breast tumor did not meet the criteria for cCR, cPR, or cP ; progressive disease (cP): there was a 50 % or greater increase in tumor size. Pathological therapeutic effect was assessed for resected primary tumors after surgery. pCR was defined as the absence of all invasive disease in the breast tumor and no residual tumor in axillary lymph nodes for histopathological therapeutic effect [19, 20].

### Clinical outcomes

A relapse event is defined as any local relapse and distant relapse including invasive ipsilateral breast tumor recurrence, ipsilateral DCIS, local invasive recurrence, regional invasive recurrence and appearance of metastases. RFS is defined as time before any relapse event according to DATECAN guidelines for breast cancer [21]. And BCSS were calculated from the date of diagnosis until the date the patient succumbed to the disease or the last follow-up time. Patients who succumbed to unrelated causes with no evidence of disease were censored.

### Follow-up

The presence of a relapse event was determined by means of imaging modalities, including CT, MRI, US, SPECT, PET-CT and biopsy of suspicious lesions. The patients underwent at least one type of imaging examination at intervals of 3–4 months during the first 2 years after surgery, and at intervals of 4–6 months thereafter until 5 years after surgery, and at intervals of 12 months after 5 years since surgery.

### Statistical analyses

The capacity of NLR in predicting relapse events was analyzed using receiver operating characteristic (ROC) curve analysis. The *T* test (or Mann-Whitney *U* test) and Wilcoxon rank sum test were used for comparing the differences of variables between two groups, when appropriate. All the continuous variables are expressed as the median (Q1 [25th percentile] - Q3 [75th percentile]) value. The association between NLR and pCR was evaluated using the chi-square test. We used the Kaplan-Meier Method and Cox proportional hazard model as univariate and multivariate analysis, respectively. In all analyses, differences were considered significant at *p* < 0.05. Statistical analyses were performed using SPSS 19.0 software (SPSS Inc, Chicago, IL).

## Results

### Patients’ features

We identified 347 patients who were diagnosed and completed the treatment for breast cancer, and 215 patients were eligible for analysis. The baseline characteristics of the study subjects are summarized in Table 1.

**Table 1:** The characteristics of 215 patients with breast cancer

Characteristic	No. (%)	
Age (yr), mean ± SD	46.41 ± 9.82	
Age (yr)		
<35	32 (14.9)	
>35	183 (85.1)	
Histology		
Ductal	198(92.1)	
Lobular	5(2.3)	
Others	12(5.6)	
T stage	c T^a^	p T^a^
T0-T1	3 (1.4)	131(60.9)
T2	108(50.2)	64(29.8)
T3	84 (39.1)	7(3.3)
T4	20 (9.3)	13(6.0)
N stage	c N^a^	p N^a^
N0	46 (21.4)	89(41.4)
N1	108 (50.2)	55(25.6)
N2	43 (20.0)	43(20.0)
N3	18 (8.4)	28(13.0)
TNM stage	cTNM stage^a^	pTNM stage^a^
0	0	26(12.1)
I	0	36(16.7)
II	108(50.2)	80(37.2)
III	107(49.8)	72(33.5)
IV	0	1(0.5)
HG		
I	68 (31.6)	
II	93 (43.3)	
III	54 (25.1)	
ER		
-	65 (30.2)	
+	150 (69.8)	
PR		
-	73 (34.0)	
+	142 (66.0)	
HER2		
-	138 (64.2)	
+	77 (35.8)	
Molecular subtype		
Luminal A	120 (55.8)	
Luminal B	52 (24.2)	
HER2-enriched	25 (11.6)	
Triple-negative	18 (8.4)	
Chemotherapy regimen		
EC	13(6.0)	
TC	18(8.4)	
TEC	161(74.9)	
TCH	23 (10.7)	
Surgery		
Breast-conserving surgery	89(41.4)	
Modified mastectomy	126(58.6)	
Chemotherapy cycles		
3	40(18.6)	
4	150(69.8)	
>4	25(11.6)	
pCR		
Yes	42(19.5)	
No	173(80.5)	
Relapse (local and distant)		
Yes	39 (18.1)	
No	176 (81.9)	
Death		
Yes	32 (14.9)	
No	183 (85.1)	
Follow-up time (months)		
Median	55.0	
Mean	57.6 ± 27.1	
Range	36.1, 75.8	

^a^cT, cN, cTNM are clinical stages before NAC. pT, pN, pTNM are pathological stages after surgery

*EC* epirubicin and cyclophosphamide, *TC* docetaxel and cyclophosphamide, *TEC* docetaxel, epirubicin and cyclophosphamide, *TCH* docetaxel, carboplatin and trastuzumab

The median value of pretreatment NLR was 2.05 (range, 0.45-15.04). Of the total of 215 patients, 111 (51.6 %) patients had NLR less than 2.1. A NLR greater than or equal to 2.1 was associated with increased T stage, TNM stage, relapse events, higher CRP value, and breast cancer specific mortality (Table 2). Therefore, patients in the higher NLR group before treatment tended to have higher staging and worse survival.

**Table 2:** Baseline characteristics by NLR

		NLR		
Characteristic	No. of patients	<2.1 (*n* = 111)	≥2.1 (*n* = 104)	*P*
		No. (%)	No. (%)	
Age (yr)^a^	215	45.4 ± 9.3	47.5 ± 10.2	NS
cT^b^	215			<0.05
1		2 (1.8)	1 (1.0)	
2		66 (59.5)	42 (40.4)	
3		36 (32.4)	48 (46.2)	
4		7 (6.3)	13 (12.5)	
cN^b^	215			NS
0		25 (22.5)	21 (20.2)	
1		61 (55.0)	47 (45.2)	
2		18 (16.2)	25 (24.0)	
3		7 (6.3)	11 (10.6)	
cTNM^b^	215			<0.05
0		0(0.0)	0(0.0)	
1		0(0.0)	0(0.0)	
2		67 (60.4)	41 (39.4)	
3		44 (39.6)	63 (60.6)	
HG	215			NS
1		36 (32.4)	32 (30.8)	
2		51 (45.9)	42 (40.4)	
3		24 (21.6)	30 (28.8)	
ER	215			NS
-		27 (24.3)	38 (36.5)	
+		84 (75.7)	66 (63.5)	
PR	215			NS
-		39 (35.1)	34 (32.7)	
+		72 (64.9)	70 (67.3)	
HER2	215			NS
-		68 (61.3)	70 (67.3)	
+		43 (38.7)	34 (32.7)	
ER^+^ and/or PR^+^	174	93 (83.8)	81 (77.9)	NS
ER^−^ PR^−^	41	18 (16.2)	23 (22.1)	
Molecular subtype	215			NS
Luminal A		62 (55.9)	58 (55.8)	
Luminal B		30 (27.0)	22 (21.2)	
HER2-enriched		12 (10.8)	13 (12.5)	
Triple-negative		7 (6.3)	11 (10.6)	
CRP (before NAC)	215	1.5 (0.5, 4.8)	2.9 (0.9, 6.7)	<0.05
Relapse (local and distant)				<0.05
No	176	98 (88.3)	78 (75.0)	
Yes	39	13 (11.7)	26 (25.0)	
Death				<0.05
No	183	102 (91.9)	81 (77.9)	
Yes	32	9 (8.1)	23 (22.1)	

^a^Mean ± SD. ^b^cT, cN, cTNM are clinical stages before NAC

ROC analysis showed that if the chosen cut-off point for NLR was 2.1, the specificity and sensitivity were 55.7 %, 66.7 %, respectively. These were statistically significant (*p* < 0.05; AUC = 0.598, 95 % CI: 0.511-0.686) (Additional file 1: Figure S1).

Higher NLR before treatment was associated with higher CRP. However, there was no significant correlation between CRP value and NLR (Pearson correlation coefficient 0.324, *p* = 0.068, Additional file 2: Figure S2).

### Association between NLR and pathologic response

**Fig. 1:**
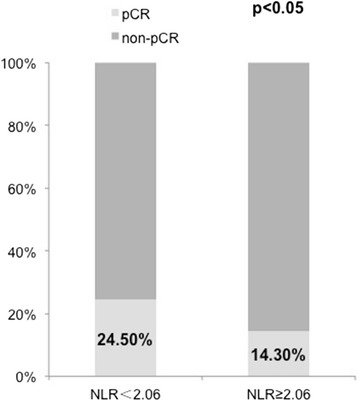
Percentages of pCR in patients stratified by NLR. In the chi-square test, the patients were divided into two groups based on the NLR cutoff (NLR < 2.06 group and NLR ≥ 2.06 group). Patients in the NLR < 2.06 group showed significantly higher pCR rate than did patients in the NLR ≥ 2.06 group (*p* < 0.05)

An increased pCR rate was observed primarily in those patients with lower NLR before treatment. The overall pCR rate was 19.5 % (42 of 215 patients). Patients in the NLR < 2.06 group showed significantly higher pCR rate than did patients in the NLR ≥ 2.06 group (NLR < 2.06 vs. NLR ≥ 2.06, 24.5 % vs. 14.3 %, *p* < 0.05, *χ*2 test) (Fig. 1).

We performed univariate and multivariate analysis including pCR with established clinicopathologic parameters. As shown in Tables 3 and 4, the percentage of pCR was a significant independent parameter, with a hazard ratio (HR) for pCR of 1.53 (95 % CI: 1.09 to 5.65, *p* < 0.05) in RFS as well as BCSS (HR: 3.37, 95 % CI: 1.93 to 28.26, *p* < 0.05) (Table 4).

**Table 3:** Hazard ratios of baseline characteristics for RFS and BCSS (univariate analysis)

		RFS		BCSS	
Variable	No.	Hazard ratio (95 % CI)	*P*	Hazard ratio (95 % CI)	*P*
Age	215	1.16 (0.46–2.98)	NS	1.11 (0.39–3.16)	NS
pT stage^a^	215				
T0		1.0		1.0	
T1		1.58 (0.46–5.46)	NS	1.54 (0.34–7.06)	NS
T2		2.12 (0.61–7.37)	NS	2.30 (0.51–10.41)	NS
T3		7.80 (1.74–34.97)	<0.01	11.29(2.06–61.89)	<0.01
T4		2.64 (0.53–13.10)	NS	8.95 (1.73–46.29)	<0.01
pN stage^a^	215				
N0		1.0		1.0	
N1		1.77 (0.66–4.74)	NS	2.38 (0.69–8.23)	NS
N2 and N3		2.75 (1.01–7.56)	<0.05	4.61 (1.32–16.08)	<0.05
pTNM^a^	215				
0–1		1.0		1.0	
2		1.15 (0.41–3.22)	NS	1.63 (0.50–5.30)	NS
3–4		3.41 (1.39–8.35)	<0.01	3.92 (1.33–11.54)	<0.05
HG	215				
1		1.0		1.0	
2		2.68 (0.56–12.89)	NS	1.65 (0.30–9.01)	NS
3		26.29(6.25–10.57)	<0.001	21.70(5.11–92.12)	<0.001
Hormone receptor	215				
ER^+^ PR^+^		1.0		1.0	
ER^+^ or PR^+^		1.88 (0.93–3.82)	NS	2.05 (0.94–4.50)	NS
ER^−^ PR^−^		1.76 (0.76–4.08)	NS	1.95 (0.77–4.89)	NS
Her2	215				
+		0.84 (0.43–1.66)	NS	1.14 (0.56–2.34)	NS
-		1.0		1.0	
Molecular subtype	215				
Luminal A		1.0		1.0	
Luminal B		1.12 (0.53–2.39)	NS	1.32 (0.58–3.00)	NS
HER2-enriched		0.81 (0.24–2.71)	NS	1.04 (0.30–3.58)	NS
Triple-negative		2.07 (0.78–5.50)	NS	2.11 (0.70–6.33)	NS
pCR	215				
Yes		1.0		1.0	
No		3.00 (1.92–9.73)	<0.05	9.05 (1.24–65.97)	<0.05
Chemotherapy regimen	215				
TEC		1.0		1.0	
TCH		0.89 (0.67-2.93)	NS	1.65 (0.54-3.42)	NS
TC		1.19 (0.86–2.25)	NS	1.32 (0.42–2.91)	NS
EC		1.23 (0.63–2.40)	NS	1.20 (0.58–2.48)	NS
Surgery	215				
Breast-conserving surgery		1.0		1.0	
Modified mastectomy		0.55 (0.27–1.10)	NS	0.46 (0.21–1.03)	NS
Chemotherapy cycles	215				
3		1.0		1.0	
4		1.15 (0.52–2.54)	NS	1.07 (0.47–2.48)	NS
>4		0.64 (0.17–2.42)	NS	0.76 (0.20–2.88)	NS
NLR	215				
NLR < 2.1		1.0		1.0	
NLR ≥ 2.1		2.11 (1.09–4.11)	<0.05	2.45 (1.13–5.31)	<0.05
CRP (before NAC)	215	1.03 (1.01–1.05)	<0.001	1.03 (1.01–1.06)	<0.01

^a^pT, pN, pTNM are pathological stages after surgery. *EC* epirubicin and cyclophosphamide, *TC* docetaxel and cyclophosphamide, *TEC* docetaxel, epirubicin and cyclophosphamide, *TCH* docetaxel, carboplatin and trastuzumab

**Table 4:** Cox proportional multivariate hazard model for relapse-free survival and breast cancer-specific survival

	RFS			BCSS	
Variable	No.	Hazard ratio (95 % CI)	*P*	Hazard ratio (95 % CI)	*P*
pTNM^a^	215				
0–1		1.0		1.0	
2		1.77 (0.89–3.53)	NS	1.36 (0.49–3.80)	NS
3–4		4.09 (1.69–9.90)	<0.05	3.37 (1.30–9.31)	<0.05
HG	215				
1		1.0		1.0	
2		2.35 (0.47–11.72)	NS	1.84 (0.32–10.52)	NS
3		26.98(5.82–125.12)	<0.001	19.21 (4.15–88.90)	<0.001
Hormone receptor	215				
ER^+^ PR^+^		1.0		1.0	
ER^+^ or PR^+^		1.53 (0.70–3.33)	NS	1.63 (0.74–3.57)	NS
ER^−^ PR^−^		3.31 (1.28–8.58)	<0.05	2.94 (1.17–7.41)	<0.05
pCR	215				
Yes		1.0		1.0	
No		1.53 (1.09–5.65)	<0.05	3.37(1.93–28.26)	<0.05
Surgery	215				
Breast-conserving surgery		1.0		1.0	
Modified mastectomy		0.80 (0.33–1.92)	NS	0.77 (0.29–2.05)	NS
NLR (before NAC)	215				
NLR < 2.1		1.0		1.0	
NLR ≥ 2.1		1.57 (1.05–3.57)	<0.05	2.21 (1.01–4.39)	<0.05
CRP (before NAC)	215	1.02 (0.99–1.05)	NS	1.00 (0.97–1.04)	NS

^a^pT, pN, pTNM are pathological stages after surgery

### Relapse-free survival and breast cancer-specific survival by NLR status

**Fig. 2:**
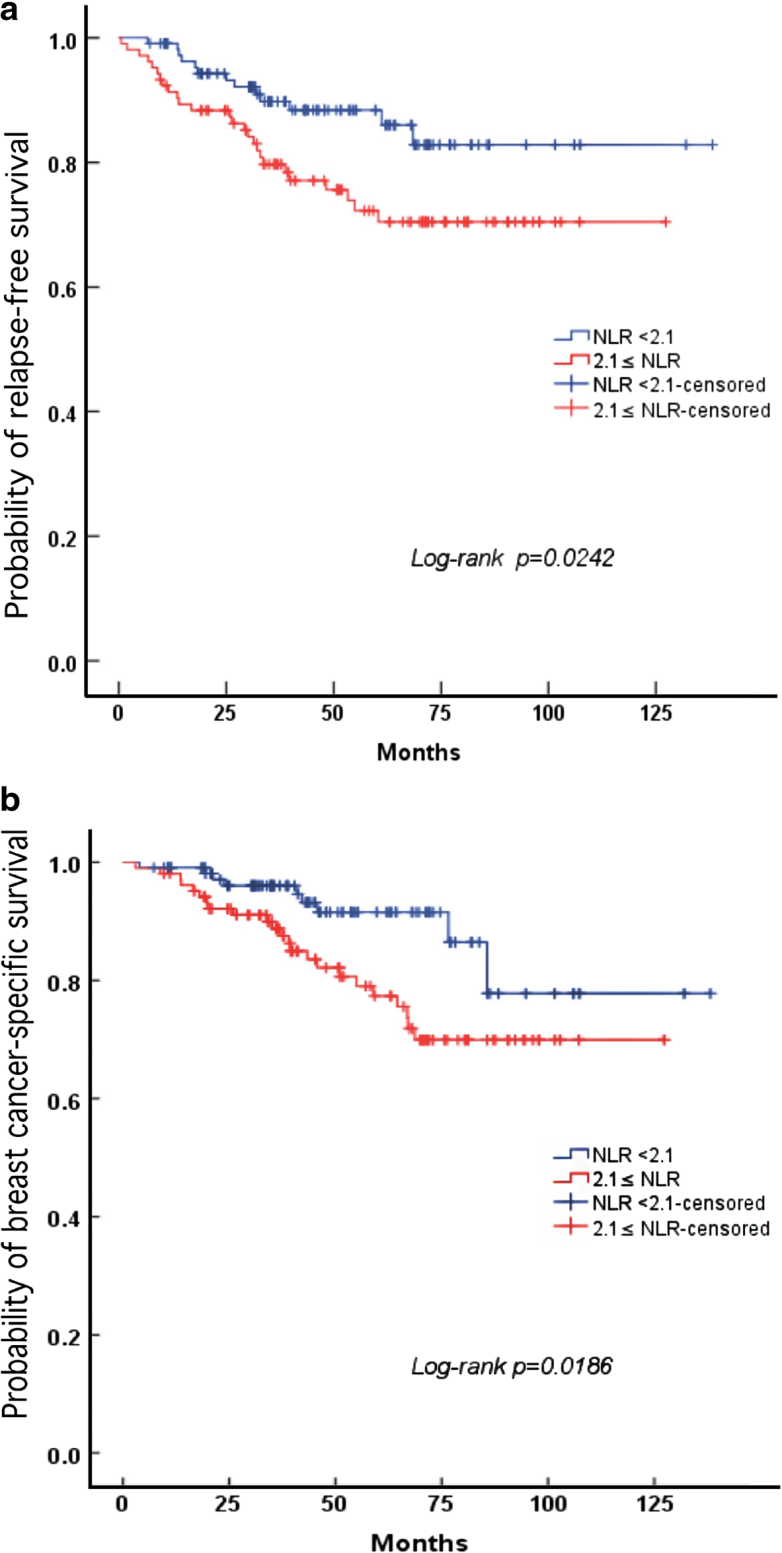
Kaplan-Meier estimates for RFS and BCSS stratified by NLR. The patients were divided into two group based on the NLR cutoff (NLR < 2.1group and NLR ≥ 2.1 group). a. Relapse-free survival in the patients based on the NLR cutoff (*p* < 0.05). b. Breast cancer-specific survival in the patients based on the NLR cutoff (*p* < 0.05)

Kaplan–Meier curves showed significantly higher (log-rank *p* < 0.05) relapse-free survival and breast cancer-specific survival in the lower NLR group before treatment (NLR < 2.1) compared with the higher NLR group (NLR ≥ 2.1) (Fig. 2).

With a median follow up of 55 months, 39 (18.1 %) and 32 (14.9 %) patients had relapse events and death events, respectively. In univariate analysis, pretreatment NLR; CRP value; advanced T, N, and AJCC stages; HG and pCR after NAC were all associated with RFS and BCSS. Higher NLR was associated with decreased RFS and BCSS (respectively: HR: 2.11, 95 % CI: 1.09-4.11, *p* < 0.05; HR: 2.45, 95 % CI: 1.13-5.31, *p* < 0.05) in our univariate analysis (Table 3). Next, pTNM stage, HG, hormone receptor, pCR, operation method, NLR and CRP were incorporated into the multivariate analysis, which further confirmed that NLR before treatment was an independent risk factor for RFS and BCSS, with respective HRs of 1.57 (95 % CI: 1.05-3.57, *p* < 0.05) and 2.21 (95 % CI: 1.01-4.39, *p* < 0.05), respectively. We did not include T-stage because there might be colinearity between T-stage and TNM-stage (Table 4).

## Discussion

In this study, we examined a cohort of breast cancer patients who received neoadjuvant chemotherapy to provide evidence on the predictive value of pathologic complete response and the prognostic value of NLR. The main finding of our analysis is that high pretreatment NLR was associated with pCR and was a significant independent predictor of RFS and BCSS in breast cancer patients undergoing preoperative chemotherapy.

To date, few studies have examined whether pretreatment NLR is predictive for pCR. Only one study has determined the relationship between pCR and pretreatment peripheral blood NLR in patients who had NAC for locally advanced BC. In that study, Eryilmaz et al. [22] showed no relationship between pCR and pretreatment NLR value, in contrast to our results. To our knowledge, this is the first time that a strong association between pretreatment NLR and chemotherapy response is described in a breast cancer study. Our results demonstrate that patients with NLR ≥ 2.06 showed poor response to neoadjuvant chemotherapy (Fig. 1). Patients with NLR < 2.06 showed a higher pCR rate than those with NLR ≥ 2.06. The major causes of these contrasting findings may be the insufficient sample size (only 78 patients) and nonstandardized therapies (some patients had anthracycline-taxane based, some had hormonal-based NACs) in the study by Eryilmaz et al. [22]. For this reason, our results are more reliable. A lower NLR value (<2.06) is more likely to reach pCR, and it is useful in consultation for patients and clinical decision-making.

Patients showing a pCR to neoadjuvant chemotherapy enjoy prolonged disease-free survival [23], which corroborates our finding that patients with NLR < 2.1 showed a relatively better prognosis. Meanwhile, an elevated pretreatment NLR is associated with worse RFS and BCSS. We found that elevated NLR at initial clinical presentation of breast cancer was an independent factor for poor survival rate in breast cancer patients. This finding is consistent with previous reports in several other cancers as well as breast cancer [6, 8–10, 24, 25]. A higher NLR (NLR > 3.3) has been correlated with an advanced stage of breast cancer [12]. Additionally, higher-NLR patients (NLR > 2.5), especially with the luminal A subtype, show significantly poorer prognosis than lower-NLR patients [13]. Previous studies included patients irrespective of whether they received NAC, whereas we only focused on patients who received NAC. Azab et al. [12] used the 75th NLR percentile as the NLR cutoff, while Noh [13] used receiver operating characteristic (ROC) curve analysis to determine the NLR cutoff. Our study also used ROC curves to determine the cutoff, and our NLR cutoff was 2.11. Regardless of these differences, the results from our study appear to favor the same conclusion: that patients with an elevated pretreatment NLR show poorer disease-specific survival than patients without elevated NLR.

The association between an elevated NLR and poor prognosis is complex. Increasing evidence suggests that cancer progression is influenced by the systemic inflammatory response [26]. Components of this inflammatory response are associated with patients’ prognostic outcomes. An elevated NLR is due to a relative neutrophilia and lymphocytopenia that occurs as part of the systemic inflammatory response triggered by cancer [27–30]. First, neutrophils may inhibit immune system function. Neutrophils promote remodeling the extracellular matrix, which promotes tumor growth and metastasis via its enzymatic actions, including the release of reactive oxygen species (ROS), nitric oxide (NO), and anginas [31–33]. In addition, relative neutrophilia enhances tumor growth and progression by activating inflammatory markers that include pro-angiogenic factors (VEGF), growth factors (CXCL8), proteases and anti-apoptotic markers (NF-kB) [9, 12, 34, 35]. In breast cancer, neutrophil-derived oncostatin M signals human breast cancer cells to secrete VEGF and increases breast cancer cells’ detachment and invasiveness [36]. On the other hand, lymphocytic response is the main component of controlling cancer progression. Increased lymphocyte infiltration has been correlated with higher pCR rate and a better prognosis in breast cancer patients who received neoadjuvant chemotherapy [37–39]. Lymphocytes (especially T4 helper and T8 suppressor lymphocytes) decline markedly in the cell-mediated immune system [29]. Moreover, immune modulators, including TGF β, IL10 and CRP, released by tumor cells impair lymphocyte action in systemic inflammation [40]. Tumor-infiltrating lymphocytes such as natural killer and T helper type 1 are effective components against cancer growth and/or metastasis in several cancers via their production of interferon gamma [41]. Chemotherapy might be an effective immunotherapy against such tumor types, and the combined effect of chemotherapeutic destruction of tumor cells and increased immune response may result in a pCR [39, 42]. Thus, a low lymphocytic infiltration at tumor margins corresponds with a poorer prognosis [27, 43, 44].

In this study, patients in the higher pretreatment NLR group tended to have higher staging. This corroborates previous reports that these preoperative characteristics are associated with vascular invasion and a more aggressive phenotype [44–46]. Stage is directly representative of tumor progression and is subsequently reflective of the immune response (neutrophilia and lymphocytopenia), and it is not surprising that higher stages correspond to higher NLR and therefore worse survival [43].

There was a significant discordance of NLR cutoffs used in previous studies [47]. Most of the studies have used an NLR of 5 as the cutoff based purely on previous work. Only four studies used ROC sensitivity and specificity analyses to determine an NLR cutoff. Azab et al. [12] used 75th NLR percentile as the NLR cutoff. Although most studies used NLR > 5 as the cutoff, this does not imply that patients with an NLR < 5 were not at an increased risk. In fact, several other studies demonstrated NLR ranges of 4 and below (even as low as 1.9) as having prognostic significance in overall survival [47]. We used ROC curve analysis to determine the NLR cutoff. ROC curve analysis suggested that the optimum NLR cut-off point was 2.11 (AUC: 0.589, 95 % CI: 0.511- 0.686, *p* < 0.05) with a sensitivity of 66.7 % and specificity of 55.7 %. Pichler et al. [48] mentioned that the ideal cutoff value for a continuous NLR was calculated by testing all possible cutoffs that would discriminate between survival and cancer-related death by Cox proportional analysis. We tested all possible cutoffs in this way from 2.0 to 2.9, and the ideal cutoff value was 2.1 for survival as well as 2.06 for pCR^+^ and pCR^−^ patients. Most studies focus on different tumors, which tend to have different inflammatory status. Even in breast cancer patients, different age, stage and phenotype correspond with different immune response and therefore different NLR.

Additionally, we are interested in the relationship between the change in NLR (ΔNLR) and its relationship with pCR or relapse-free survival. We found no significance in the relationship between ΔNLR and pCR or RFS (data not shown). Different chemotherapy regimens may lead to different degrees of neutropenia, as anthracycline and taxane-based regimens can cause severe neutropenia. Patients with neutropenia after NAC were suggested to take granulocyte colony-stimulating factors (G-CSF) to stimulate the release of leucocytes, which may also have affected neutrophil and lymphocyte counts. That would result in different baseline NLR after NAC. So we believe that the pretreatment NLR is likely to be the most robust NLR value to use.

The major limitation of our study is the retrospective nature. Many patients whose records lacked information or who were lost to follow-up were not enrolled in the study, and that may have led to selection bias. Second, it was beyond the scope of this study to make clear whether patients with Her-2 positive tumors had taken Herceptin as adjuvant treatment because not all the patients could afford the high price before 2010 in China. This might have had some statistical influence on survival because Herceptin has made such an enormous impact, particularly on disease-free survival. Third, patients with different ages, stages and phenotypes corresponded to different immune responses, and we were not able to conduct a stratified analysis on such small subgroups of patients. Moreover, our study lacked any evaluation of tumor-associated neutrophils and lymphocytes. Furthermore, analysis about local recurrence-free survival and metastasis-free survival relating to long-term outcome were limited by the patients’ records. Besides, further study into the relationship between tumor-infiltrating lymphocytes and NLR is needed to validate our results. The aforementioned limitations taken together with the relatively small sample size suggest that our results need to be validated in additional independent cohorts of breast cancer patients, ideally through large-scale prospective clinical studies.

Pretreatment NLR represents a simpler, more robust and more convenient parameter compared with other pathological indicators, such as KI67. The use of pretreatment NLR may facilitate the administration of NAC therapy in patients with lower NLR to reach a better pCR rate and to enhance long-term outcomes.

## Conclusions

Our findings suggest that NLR is an important factor predicting the response to NAC in breast cancer patients. Patients with higher NLR showed a lower percentage of pCR after NAC, and high NLR was an independent significant predictor of lower RFS and BCSS in breast cancer patients. Further prospective, multicenter studies are needed to validate our results.

### Ethics approval and consent to participate

The study was given ethical approval with Ethical Committee of Sun Yat-sen Memorial Hospital and all the patients had given written informed consent.

### Consent for publication

Not applicable.

## Availability of data and materials

The dataset surpporting the conclusions of this article is available in the LabArchives [http://labarchives.com/bmc] repository [https://mynotebook.labarchives.com/share/chenxixi/MjAuOHwxNzc5MzMvMTYvVHJlZU5vZGUvMjMxMjM0NDU5MHw1Mi44].

## Additional files

Additional file 1: Figure S1. Assessment of cutoff value of NLR for prediction of relapse events with ROC curve analysis. ROC analysis showed that if the chosen cut-off point for NLR was 2.1, the specificity and sensitivity were 55.7 %, 66.7 %, respectively. These were statistically significant (*p* < 0.05; AUC = 0.598, 95 % CI: 0.511-0.686) (TIFF 2566 kb) Additional file 2: Figure S2. Relationship between CRP value and NLR. The x-axis indicates the CRP value and the y-axis shows the value of NLR. The relationship was investigated using Pearson’s correlation coefficient test (*p* **=** 0.068). (TIFF 9028 kb)

## Abbreviations

BCSS: breast cancer-specific survival
CRP: C reactive protein
ER: estrogen receptor
HER2: human epidermal growth factor receptor 2
HG: histologic grade
NAC: neoadjuvant chemotherapy
NLR: neutrophil-to-lymphocyte ratio
pCR: pathologic complete response
PR: progesterone receptor
RFS: relapse-free survival
