# Supplementary figures and images for: Pretreatment neutrophil-to-lymphocyte ratio is correlated with response to neoadjuvant chemotherapy as an independent prognostic indicator in breast cancer patients: a retrospective study
Source: BMC Cancer. 2016 May 19;16:320. doi: 10.1186/s12885-016-2352-8 (PMC4872336; doi:10.1186/s12885-016-2352-8)

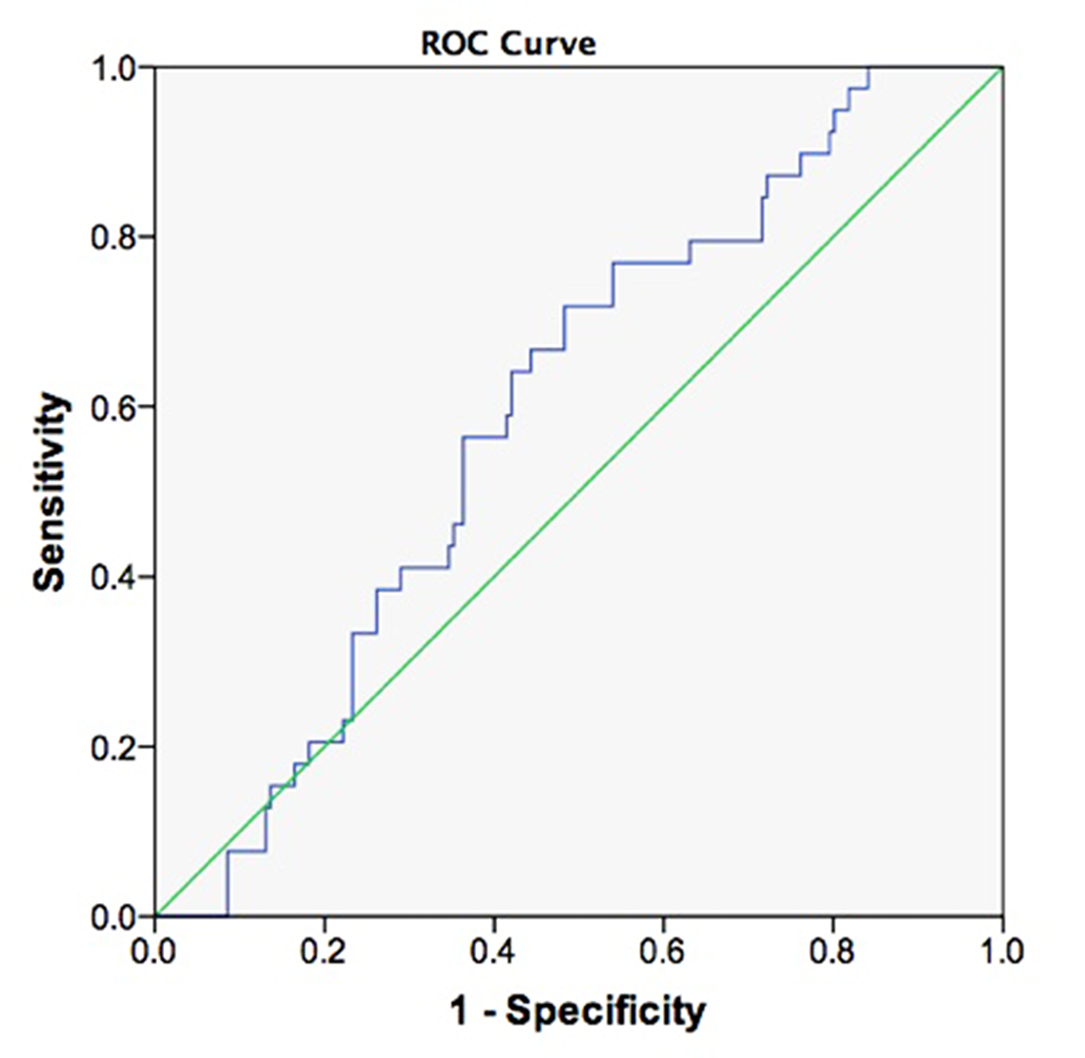

Supplement: Additional file 1: Figure S1. — Assessment of cutoff value of NLR for prediction of relapse events with ROC curve analysis. ROC analysis showed that if the chosen cut-off point for NLR was 2.1, the specificity and sensitivity were 55.7 %, 66.7 %, respectively. These were statistically significant (p < 0.05; AUC = 0.598, 95 % CI: 0.511-0.686) (TIFF 2566 kb) [file 12885_2016_2352_MOESM1_ESM.tiff]

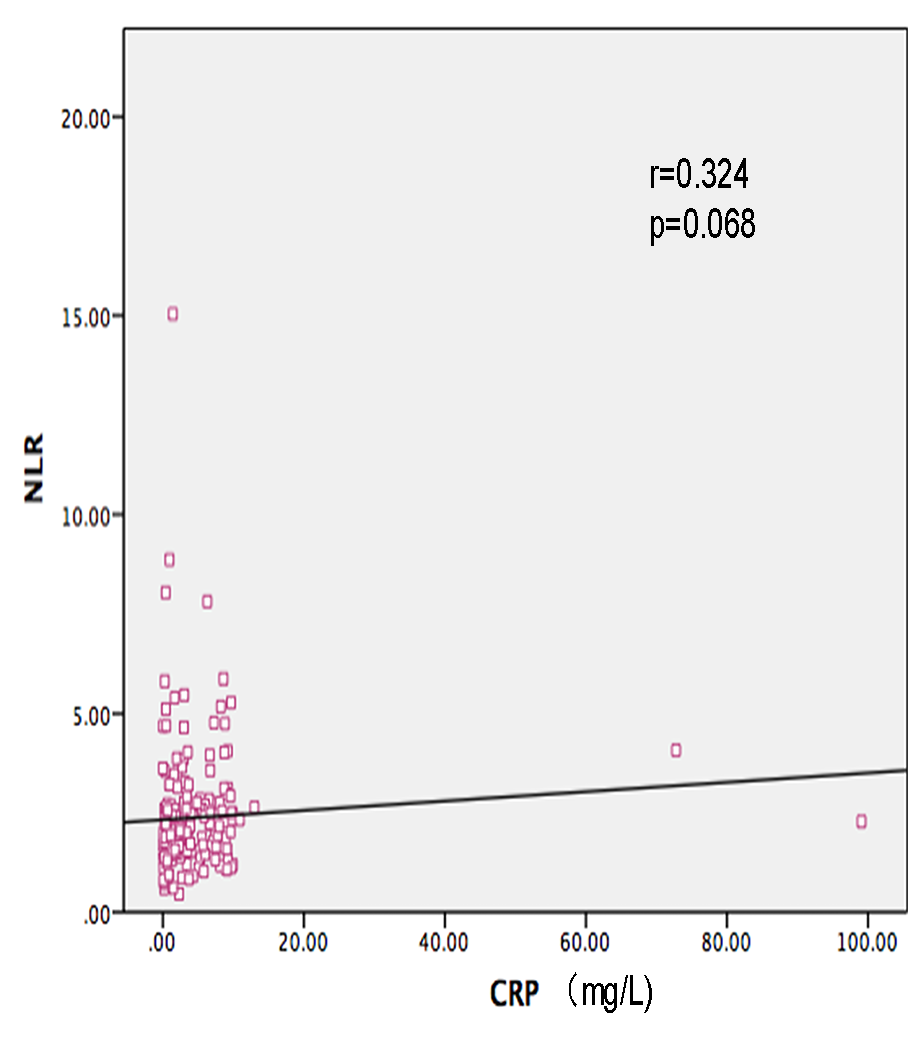

Supplement: Additional file 2: Figure S2. — Relationship between CRP value and NLR. The x-axis indicates the CRP value and the y-axis shows the value of NLR. The relationship was investigated using Pearson’s correlation coefficient test (p = 0.068). (TIFF 9028 kb) [file 12885_2016_2352_MOESM2_ESM.tiff]
